# Characteristics and diagnostic performance of surface electromyography in individuals with cognitive impairment

**DOI:** 10.1002/alz70856_100146

**Published:** 2025-12-25

**Authors:** Xiaoli Hao, Xuan Yang, Lu Shen, Bin Jiao

**Affiliations:** ^1^ Xiangya Hospital, Central South University, Changsha, Hunan, China

## Abstract

**Background:**

Motor changes precede the emergence of cognitive impairment (CI) and surface electromyography (sEMG) is a non‐invasive and convenient tool. The ability of sEMG for CI detection remain unclear.

**Method:**

639 participants were enrolled, including 284 patients with CI, and 355 controls. A series of motor and cognitive assessment were conducted. The sEMG examination under single and dual‐task patterns were performed. The plasma phosphorylated tau 181(*p*‐tau181) was measured in a subset group.

**Result:**

The CI group exhibited significantly decreased motor function, including muscle bulk, swallowing, balance, and gait. Significant alterations of 20 sEMG features were detected in CI group compared to controls (*p* <0.05), among them, the integrated electromyography feature of right gastrocnemius lateralis in dual‐task was positively associated with plasma *p*‐tau181. Furthermore, the combination of single and dual‐task sEMG model can effectively distinguish CI and MCI from controls with an area under the curve (AUC) of 0.849 and 0.875 respectively, and the AUC for CI detection reached 0.948 after combining with *p*‐tau181.

**Conclusion:**

Patients with CI always coexist with motor change, and sEMG can serve as an effective tool for CI screening.

